# The Value of Circulating Nogo-B for Evaluating Hepatic Functional Reserve in Patients with Cirrhosis

**DOI:** 10.1155/2015/419124

**Published:** 2015-05-06

**Authors:** Maoyao Wen, Ruoting Men, Zongze Yang, Xuelian Dan, Wenchao Wu, Xiaojing Liu, Li Yang

**Affiliations:** ^1^Division of Digestive Diseases, West China Hospital of Sichuan University, Chengdu, Sichuan 610041, China; ^2^Creation and Management of a Tumour Bank, West China Hospital of Sichuan University, Chengdu, Sichuan 610041, China; ^3^Laboratory of Cardiovascular Diseases, Regenerative Medicine Research Center, West China Hospital of Sichuan University, Chengdu, Sichuan 610041, China

## Abstract

*Objective*. To examine Nogo-B in liver tissues and plasma of patients with liver cirrhosis and associate them with various clinical parameters. *Materials and Methods*. Nogo-B protein expression was examined by immunohistochemistry in 24 human fibrotic/cirrhotic liver specimens and 10 healthy controls. We determined plasma Nogo-B levels by enzyme-linked immunosorbent assay in 301 patients with liver cirrhosis and 153 healthy controls, and then analyzed various clinical parameters. *Results*. Nogo-B was mainly expressed in nonparenchymal cells in the liver and was marked increased in liver with significant fibrosis/cirrhosis compared to controls. Moreover, Metavir F4 showed a higher level of expression than F2. Plasma Nogo-B levels were significantly higher in cirrhotic patients than in healthy controls and were the highest in Child-Pugh class C patients. Plasma Nogo-B levels were positively correlated with Child-Pugh scores. However, there was no relationship between plasma Nogo-B levels and etiology of liver diseases, ALT, AST, platelet counts, and the severity of esophagogastric varices. *Conclusions*. Nogo-B is mainly expressed in hepatic nonparenchymal cells and is present in plasma. Abnormally high plasma levels of Nogo-B are associated with hepatic cirrhosis and Child-Pugh score, but not correlated with the grade of liver inflammation or portal hypertension. Plasma Nogo-B may be a novel surrogate marker to reflect liver function reserve.

## 1. Introduction

Liver fibrosis/cirrhosis is a major cause of mortality around the world and the development of cirrhosis has been considered to be an irreversible event [1, 2]. Prognosis of patients with liver cirrhosis often depends on their hepatic functional reserve. Blood tests play pivotal roles in the clinical assessment of liver function reserve. Clinical classification systems, such as model for end-stage liver disease (MELD) and Child-Pugh scoring systems, are widely used. Even radiographic examination of the remnant liver volumes can be helpful [3]. It is desirable to have a simple noninvasive blood test that reflects both the stage of liver fibrosis and functional reserve.

Nogo-B is a member of the reticulum (Rtn) family of proteins localized primarily in the endoplasmic reticulum (ER) and is widely distributed in cardiac myocyte, vascular endothelial cell, smooth muscle cell, testis, and other tissues [4–6]. Recent studies have shown that Nogo-B levels are significantly elevated in rat with cirrhosis, whereas low levels of Nogo-B suggest the absence of liver fibrosis [7]. In our previous study, we found that the plasma Nogo-B levels in patients with hepatic cirrhosis were significantly higher than healthy controls [8]. As a potential indicator of hepatic cirrhosis, the correlation between Nogo-B and clinical characteristics of cirrhosis remains unclear. Thus, we examined Nogo-B protein expression in fibrotic/cirrhotic liver tissues by immunohistochemistry and further investigated hepatic cirrhotic patients with different etiology and severity. Then we analyzed the relationship between plasma Nogo-B levels and the commonly used clinical parameters of liver function in all patients.

## 2. Methods

### 2.1. Subjects

Fibrotic/cirrhotic liver specimens were obtained from 24 patients who underwent liver surgery for a variety of liver diseases. Twelve of the specimens showed Metavir stage 2 (F2) and the other 12 showed stage 4 (F4) fibrosis. The 10 healthy liver specimens were from patients who underwent liver surgery for benign focal lesions [9]. All the liver specimens were provided by the Department of Pathology, West China Hospital, Sichuan University. For plasma Nogo-B analysis, 301 patients of hepatic cirrhosis were enrolled in the Division of Digestive Diseases, West China Hospital, Sichuan University, from March 2012 to March 2014. 152 patients had hepatitis B cirrhosis, 83 were alcoholic cirrhosis, and 66 were primary biliary cirrhosis (PBC). The diagnosis of cirrhosis was made based on imaging studies and impaired liver synthetic function [10]. Patients with cardiovascular disease, kidney diseases, central nervous system disorder, chronic obstructive pulmonary diseases (COPD), and pulmonary artery hypertension which might affect plasma Nogo-B levels were excluded [11, 12]. The normal control group was composed of 153 healthy volunteers. All subjects provided written informed consents. This study was approved by the medical ethics committee of the West China Hospital, Sichuan University.

### 2.2. Collection of Samples and Clinical Parameters

Blood samples from patients and healthy controls were obtained. The plasma was immediately separated by centrifugation at 800 g for 10 minutes at room temperature and stored at −80°C. Parameters of liver function, such as alanine aminotransferase (ALT), aspartate aminotransferase (AST), and platelet (PLT), were collected from the Department of Clinical Laboratory, West China Hospital, Sichuan University. The volume of ascites was observed by ultrasound. Patient's Child-Pugh class/score were calculated. Esophageal and gastric varices were screened by esophagogastroduodenoscopy. The varices were defined as either large (>5 mm) or small (<5 mm) [13].

### 2.3. Plasma Nogo-B Assay

The plasma Nogo-B levels were determined by enzyme-linked immunosorbent assay (ELISA) kit (Biolegend Inc., USA), and the assays were performed by following the manufacture's instruction.

### 2.4. Histological Analyses

Five-micrometer sections of human liver tissue were prepared and embedded in paraffin. Then these samples were stained with hematoxylin and eosin.

### 2.5. Immunohistochemistry

The sections were deparaffinized in xylene and dehydrated in ethanol and then treated with 0.01 mol/L citrate buffer of 40 minutes, pH 6.0, at 95°C in water bath. Endogenous peroxidase activity was blocked by incubation in 3% H_2_O_2_ for 15 minutes in the dark. Slides were then incubated in 1 : 200 rabbit polyclonal Nogo-A+B antibody (Abcam; ab47085) for 45 minutes at 37°C and incubated in 50–100 *μ*L ChemMate Envision+HRP (Dako; EnvisionTM Detection Kit; number K5007) for 45 minutes at 37°C. Next, diaminobenzidine (DAB) was added to each slide for coloration. Distilled water was used to end the chromogenic reaction. The slides were counterstained with hematoxylin.

### 2.6. Statistical Analysis

Statistical analyses were performed using SPSS 17.0. *P* < 0.05 was considered significant. Data were presented as mean ± standard deviations (SD) or range. Correlations were analyzed with the Spearman correlation coefficient. Multiple comparisons for different groups were carried out using unpaired *t*-test or one-way analysis of variance (ANOVA) followed by S.N.K. test. IPP 6.0 was used to analyze the results of immunohistochemistry.

## 3. Results

### 3.1. Nogo-B Is Highly Expressed in Liver Tissues with Metavir Stage F4

**Figure 1:**
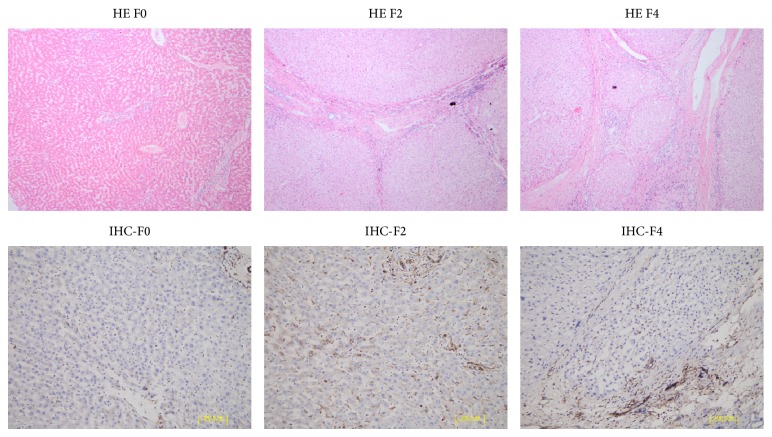
Nogo-B is associated with Metavir fibrosis scoring. Human liver tissues were stained with hematoxylin and eosin (200x) and Nogo-B is highly expressed in cirrhotic human livers (F4) and localizes primarily in nonparenchymal cells. F0: Metavir stage 0, F2: Metavir stage F2, and F4: Metavir stage F4.

Nogo-B was mainly expressed in hepatic nonparenchymal cells and highly expressed in fibrotic/cirrhotic tissues. Nogo-B expression in the liver positively correlated with the stage of liver fibrosis (Figure 1 F4* versus* F2, *P* < 0.05).

### 3.2. Circulating Nogo-B Levels Are Significantly Increased in Patients with Hepatic Cirrhosis

**Figure 2:**
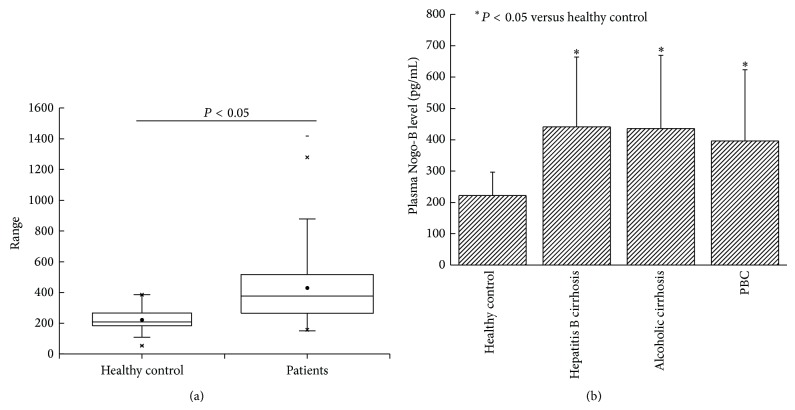
(a) Plasma Nogo-B levels were significantly higher in cirrhotic patients than in healthy controls (*P* < 0.05). (b) Plasma Nogo-B levels in patients with hepatitis B cirrhosis, alcoholic cirrhosis, and primary biliary cirrhosis were significantly higher than healthy controls while there is no significant difference among patients with different etiologies. PBC: primary biliary cirrhosis.

Clinical characteristics of patients in our study were summarized in Table 1. Our results showed that plasma Nogo-B levels were significantly higher in cirrhotic patients (429.84 ± 226.66 pg/mL) than in healthy controls (222.04 ± 74.54 pg/mL, *P* < 0.05, Figure 2(a)). The mean plasma Nogo-B levels in patients with hepatitis B cirrhosis (441.18 ± 222.69 pg/mL), alcoholic cirrhosis (435.78 ± 233.68 pg/mL), and PBC (396.28 ± 227.00 pg/mL) were not significantly different. However they were all much higher than healthy controls (222.04 ± 74.54 pg/mL, *P* < 0.05, Figure 2(b)).

### 3.3. Circulating Nogo-B Is Correlated with Child-Pugh Classification in Liver Cirrhotic Patients

**Figure 3:**
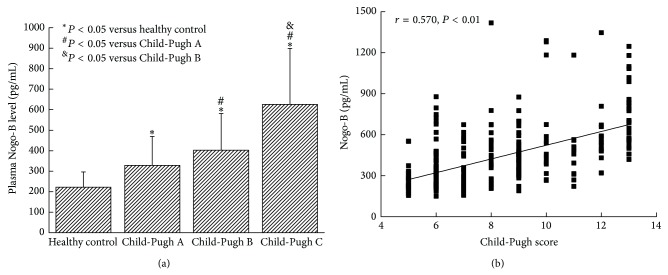
(a) Plasma Nogo-B levels were significantly higher in Child-Pugh C than Child-Pugh A and B groups (*P* < 0.05). And Nogo-B levels were significantly increased in Child-Pugh class B compared with Child-Pugh class A (*P* < 0.05). (b) Child-Pugh scores were positively correlated with plasma Nogo-B levels (*r* = 0.570, *P* < 0.01).

Child-Pugh classification is considered an important indicator to reflect the severity of liver diseases [13]. To investigate whether plasma Nogo-B levels correlate with Child-Pugh classification, we divided all 301 patients into three groups according to their Child-Pugh scores (112 patients were Child-Pugh A, 115 were Child-Pugh B, and 74 were Child-Pugh C, Table 1). The results showed that plasma Nogo-B levels were significantly higher in Child-Pugh A, B, and C groups compared with healthy controls (328.44 ± 141.17 pg/mL, 402.51 ± 179.33 pg/mL, and 625.79 ± 273.36 pg/mL* versus *222.04 ± 74.54 pg/mL, resp., *P* < 0.05). In Child-Pugh class C group, the plasma Nogo-B levels were significantly higher than Child-Pugh A and B groups (625.79 ± 273.36 pg/mL* versus *328.44 ± 141.17 pg/mL, 402.51 ± 179.33 pg/mL, resp., *P* < 0.05). And Nogo-B levels were significantly increased in Child-Pugh class B patients compared with Child-Pugh class A (*P* < 0.05, Figure 3(a)). Furthermore, the Child-Pugh scores were positively correlated with plasma Nogo-B levels (*r* = 0.570, *P* < 0.01, Figure 3(b)).

### 3.4. Association of Circulating Nogo-B Levels with Alanine Aminotransferase and Aspartate Aminotransferase

**Figure 4:**
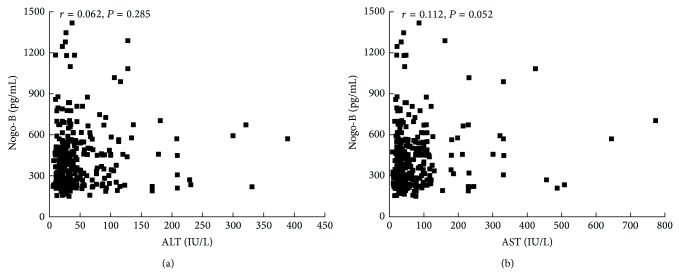
(a) Plasma Nogo-B levels were not in correlation with alanine aminotransferase (*r* = 0.062, *r* = 0.570, *P* = 0.285) and (b) aspartate aminotransferase (*r* = 0.112, *P* = 0.052). ALT: alanine aminotransferase and AST: aspartate aminotransferase.

We examined whether plasma Nogo-B levels correlated with liver inflammatory markers. The results showed no significant correlation between plasma Nogo-B concentrations and ALT (*r* = 0.062, *P* = 0.285, Figure 4(a)) or AST (*r* = 0.112, *P* = 0.052, Figure 4(b)).

### 3.5. Circulating Nogo-B and the Relationship with the Platelet Counts and the Degree of Esophageal and Gastric Varices

**Figure 5:**
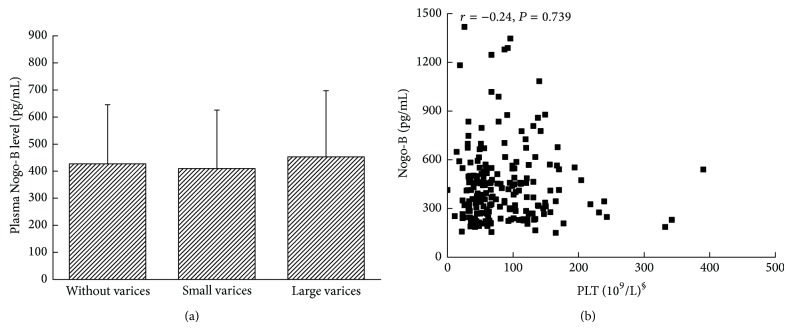
(a) Plasma Nogo-B levels have no significant changes in patients with different degrees of esophageal and gastric varices. (b) Platelet counts in patients who did not receive splenic embolization or splenectomy were not correlated with plasma Nogo-B (*r* = −0.24, *P* = 0.739). PLT: platelet.

We analyzed the relationship between plasma Nogo-B levels and PLT counts (excluding patients with splenic embolization and splenectomy), the degree of esophageal and gastric varices. This analysis showed no significant change in patients with different degrees of esophageal and gastric varices (Figure 5(a)). PLT counts in patients who did not receive splenic embolization or splenectomy were analyzed as well, and similarly, no correlation was found (*r* = −0.24, *P* = 0.739, Figure 5(b)).

## 4. Discussion

Our study confirmed that Nogo-B protein was mainly expressed in nonparenchymal cell in liver tissues [7] and showed that the expression of Nogo-B in liver tissues positively correlated with Metavir fibrosis scoring. This result suggested that Nogo-B correlates with the histological severity of hepatic cirrhosis.

In order to further examine the relationship of Nogo-B and hepatic cirrhosis, we measured the plasma Nogo-B levels in 301 patients with hepatic cirrhosis and 153 healthy controls. We found that plasma Nogo-B levels were significantly higher in the cirrhotic patients than in controls [8]. However, there was no significant difference of plasma Nogo-B among cirrhotic patients of different etiologies (hepatitis B, alcoholic cirrhosis, and PBC). We also found a positive correlation between the plasma Nogo-B levels and the Child-Pugh scores. Child-Pugh scores were considered an important indicator of the liver functional reserve. Thus the plasma Nogo-B might be a good surrogate marker for evaluation of liver functional reserve. We found no correlation between plasma Nogo-B and ALT and AST, suggesting that plasma Nogo-B concentrations do reflect inflammation of liver. This is understandable as Nogo-B is not produced in hepatocytes. Also, Nogo-B levels were not correlated with thrombocytopenia or the severity of esophagogastric varices, suggesting that plasma Nogo-B does not reflect the severity of portal hypertension. These findings suggest that plasma Nogo-B was closely related to the degree of liver fibrosis and liver reserve function but not to the active inflammation of the liver. Due to the easy ELISA assay, Nogo-B may be used as a simple clinical test for staging liver fibrosis and assessing liver functional reserve in both liver transplantation and hepatology clinics.

In conclusion, our study showed Nogo-B was mainly expressed in nonparenchymal cells in the liver and the tissue Nogo-B levels correlated with the severity of liver fibrosis scores histologically. The plasma Nogo-B levels were significantly higher in cirrhosis patients and there was a moderate degree of correlation between the plasma Nogo-B levels and the Child-Pugh scores. Nogo-B levels however do not correlate with inflammation of liver and the severity of portal hypertension. These findings suggest that plasma Nogo-B may be a very useful surrogate marker for clinical assessment of liver fibrosis and liver function reserve in patients with chronic liver diseases with or without portal hypertension and thrombocytopenia.

## Figures and Tables

**Table 1:** Clinical data of hepatic cirrhotic patients.

Data	Value^†^
Characteristics	
Gender (male/female)	197/104
Age	51.65 (21–80)
Etiology (chronic hepatitis B infection/alcoholic cirrhosis/PBC)	152/83/66
Parameters	
Child-Pugh category (A/B/C)	112/115/74
Degrees of esophageal and gastric varices (absent/small/large)^‡^	99/102/100
*Biochemical parameters *	
Total bilirubin (*μ*mol/L)	6.1–607.7
ALT (IU/L)	6–389
AST (IU/L)	9–773
Albumin (g/L)	17.3–45.3
INR	0.89–2.28
PLT (10^9^/L)^§^	11–390

^†^Data are presented as the range or as the number of patients.

^‡^Esophageal and gastric varices were diagnosed by esophagogastroduodenoscopy, Doi: 10.1002/hep.21907.

^§^PLT was analyzed in 203 patients who were without splenic embolization and splenectomy.

ALT: alanine aminotransferase; AST: aspartate aminotransferase; INR: international normalized ratio; PBC: primary biliary cirrhosis; PLT: platelet.
